# 4-Chloro-*N*-(2-methyl­benzo­yl)benzene­sulfonamide

**DOI:** 10.1107/S1600536810050087

**Published:** 2010-12-04

**Authors:** P. A. Suchetan, Sabine Foro, B. Thimme Gowda

**Affiliations:** a Department of Chemistry, Mangalore University, Mangalagangotri 574 199, Mangalore, India; b Institute of Materials Science, Darmstadt University of Technology, Petersenstrasse 23, D-64287 Darmstadt, Germany

## Abstract

In the title compound, C_14_H_12_ClNO_3_S, the conformation of the N—H bond in the C—SO_2_—NH—C(O) segment is *anti* to the C=O bond. The two aromatic rings are tilted relative to each other by 57.7 (1)°. In the crystal, mol­ecules are linked by pairs of N—H⋯O(S) hydrogen bonds, forming centrosymmetric dimers.

## Related literature

For background to our study of the effect of ring and side-chain substituents on the crystal structures of *N*-aromatic sulfonamides and for similar structures, see: Gowda *et al.* (2010**a* ▶,b* ▶); Suchetan *et al.* (2010 ▶).
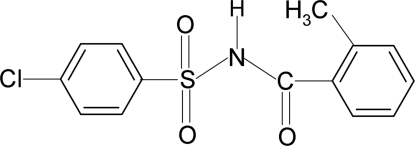


## Experimental

### 

#### Crystal data

C_14_H_12_ClNO_3_S
*M* *_r_* = 309.76
Triclinic, 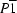

*a* = 6.4328 (9) Å
*b* = 10.343 (1) Å
*c* = 11.207 (1) Å
α = 78.78 (1)°
β = 73.84 (1)°
γ = 84.62 (1)°
*V* = 701.88 (13) Å^3^
*Z* = 2
Cu *K*α radiation
μ = 3.86 mm^−1^
*T* = 299 K
0.40 × 0.15 × 0.15 mm

#### Data collection

Enraf–Nonius CAD-4 diffractometer
4317 measured reflections
2502 independent reflections
2283 reflections with *I* > 2σ(*I*)
*R*_int_ = 0.039
3 standard reflections every 120 min  intensity decay: 0.5%

#### Refinement

*R*[*F* ^2^ > 2σ(*F* ^2^)] = 0.052
*wR*(*F* ^2^) = 0.147
*S* = 1.04
2502 reflections
186 parameters
1 restraint
H atoms treated by a mixture of independent and constrained refinement
Δρ_max_ = 0.76 e Å^−3^
Δρ_min_ = −0.56 e Å^−3^

### 

Data collection: *CAD-4-PC* (Enraf–Nonius, 1996 ▶); cell refinement: *CAD-4-PC*; data reduction: *REDU4* (Stoe & Cie, 1987 ▶); program(s) used to solve structure: *SHELXS97* (Sheldrick, 2008 ▶); program(s) used to refine structure: *SHELXL97* (Sheldrick, 2008 ▶); molecular graphics: *PLATON* (Spek, 2009 ▶); software used to prepare material for publication: *SHELXL97*.

## Supplementary Material

Crystal structure: contains datablocks I, global. DOI: 10.1107/S1600536810050087/bt5427sup1.cif

Structure factors: contains datablocks I. DOI: 10.1107/S1600536810050087/bt5427Isup2.hkl

Additional supplementary materials:  crystallographic information; 3D view; checkCIF report

## Figures and Tables

**Table 1:** Hydrogen-bond geometry (Å, °)

*D*—H⋯*A*	*D*—H	H⋯*A*	*D*⋯*A*	*D*—H⋯*A*
N1—H1*N*⋯O1^i^	0.82 (2)	2.15 (2)	2.925 (3)	157 (3)

Symmetry code: (i) 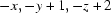.

**Fig. 1.**
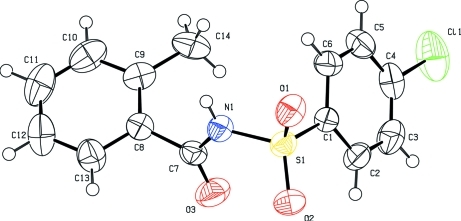
Molecular structure of the title compound, showing the atom-labelling scheme. Displacement ellipsoids are drawn at the 50% probability level.

**Fig. 2.**
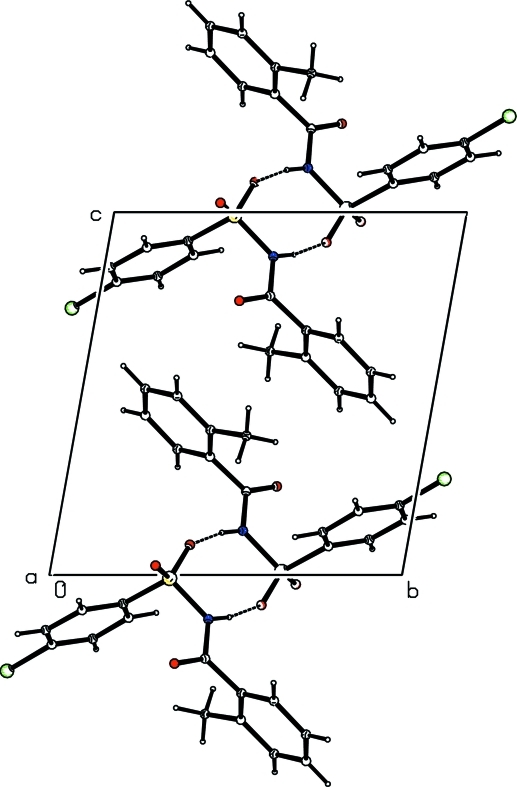
Molecular packing in the title compound. Hydrogen bonds are shown as dashed lines.

## supplementary crystallographic information

### Comment

As a part of studying the effect of ring and the side chain substituents on the crystal structures of *N*-aromatic sulfonamides (Gowda *et al.*, 2010**a*,b*; Suchetan *et al.*, 2010), the structure of 4-Chloro-*N*-(2-methylbenzoyl)benzenesulfonamide (I) has been determined. The conformation of the N—H bond in the C—SO_2_—NH—C(O) segment is *anti* to the C=O bond (Fig. 1), similar to those observed in *N*-(2-chlorobenzoyl)-4-chlorobenzenesulfonamide (II)(Gowda *et al.*, 2010*a*), *N*-(2-methylbenzoyl)-4-methylbenzenesulfonamide (III) (Gowda *et al.*, 2010*b*) and *N*-(2-methylbenzoyl)- 2-chlorobenzenesulfonamide (IV) (Suchetan *et al.*, 2010).

The molecules are twisted at the *S* atom with the torsional angle of -69.2 (2)°, compared to those of 65.7 (2)° in (II), 67.7 (2)° in (III) and -64.0 (2)° in (IV).

The dihedral angles between the sulfonyl benzene ring and the —SO_2_—NH—C—O segment is 87.2 (1)°, compared to the values of 88.5 (1)° in (II), 87.1 (1)° in (III) and 88.4 (1)° in (IV).

Furthermore, the dihedral angle between the sulfonyl and the benzoyl benzene rings is 57.7 (1)°, compared to the values of 58.0 (1)° in (II), 58.2 (1)° in (III) and 78.7 (1)° in (IV)

The packing of molecules linked by of N—H···O(S) hydrogen bonds (Table 1) is shown in Fig. 2.

### Experimental

The title compound was prepared by refluxing a mixture of 2-methylbenzoic acid, 4-chlorobenzenesulfonamide and phosphorous oxy chloride for 3 h on a water bath. The resultant mixture was cooled and poured into ice cold water. The solid, *N*-(2-methylbenzoyl)-4-chlorobenzenesulfonamide obtained was filtered, washed thoroughly with water and then dissolved in sodium bicarbonate solution. The compound was later reprecipitated by acidifying the filtered solution with dilute HCl. It was filtered, dried and recrystallized.

Rod like colourless single crystals of the title compound used in X-ray diffraction studies were obtained by a slow evaporation of its toluene solution at room temperature.

### Refinement

The H atom of the NH group was located in a difference map and refined with the N—H distance  restrained to 0.86 (2) Å. The other H atoms were positioned with idealized geometry using a riding model with C—H = 0.93–0.96 Å. All H atoms were refined with isotropic displacement parameters set to 1.2 times of the *U*_eq_ of the parent atom.

### Crystal data

**Table:**

C_14_H_12_ClNO_3_S	*Z* = 2
*M**_r_* = 309.76	*F*(000) = 320
Triclinic, *P*1	*D*_x_ = 1.466 Mg m^−^^3^
Hall symbol: -P 1	Cu *K*α radiation, λ = 1.54180 Å
*a* = 6.4328 (9) Å	Cell parameters from 25 reflections
*b* = 10.343 (1) Å	θ = 4.2–22.5°
*c* = 11.207 (1) Å	µ = 3.86 mm^−^^1^
α = 78.78 (1)°	*T* = 299 K
β = 73.84 (1)°	Rod, colourless
γ = 84.62 (1)°	0.40 × 0.15 × 0.15 mm
*V* = 701.88 (13) Å^3^	

### Data collection

**Table:**

Enraf–Nonius CAD-4 diffractometer	*R*_int_ = 0.039
Radiation source: fine-focus sealed tube	θ_max_ = 66.9°, θ_min_ = 4.2°
graphite	*h* = −7→4
ω/2θ scans	*k* = −12→12
4317 measured reflections	*l* = −13→13
2502 independent reflections	3 standard reflections every 120 min
2283 reflections with *I* > 2σ(*I*)	intensity decay: 0.5%

### Refinement

**Table:**

Refinement on *F*^2^	Secondary atom site location: difference Fourier map
Least-squares matrix: full	Hydrogen site location: inferred from neighbouring sites
*R*[*F*^2^ > 2σ(*F*^2^)] = 0.052	H atoms treated by a mixture of independent and constrained refinement
*wR*(*F*^2^) = 0.147	*w* = 1/[σ^2^(*F*_o_^2^) + (0.1004*P*)^2^ + 0.2209*P*] where *P* = (*F*_o_^2^ + 2*F*_c_^2^)/3
*S* = 1.04	(Δ/σ)_max_ < 0.001
2502 reflections	Δρ_max_ = 0.76 e Å^−^^3^
186 parameters	Δρ_min_ = −0.56 e Å^−^^3^
1 restraint	Extinction correction: *SHELXL97* (Sheldrick, 2008), Fc^*^=kFc[1+0.001xFc^2^λ^3^/sin(2θ)]^-1/4^
Primary atom site location: structure-invariant direct methods	Extinction coefficient: 0.038 (3)

### Special details

**Table:**

Geometry. All e.s.d.'s (except the e.s.d. in the dihedral angle between two l.s. planes) are estimated using the full covariance matrix. The cell e.s.d.'s are taken into account individually in the estimation of e.s.d.'s in distances, angles and torsion angles; correlations between e.s.d.'s in cell parameters are only used when they are defined by crystal symmetry. An approximate (isotropic) treatment of cell e.s.d.'s is used for estimating e.s.d.'s involving l.s. planes.
Refinement. Refinement of *F*^2^ against ALL reflections. The weighted *R*-factor *wR* and goodness of fit *S* are based on *F*^2^, conventional *R*-factors *R* are based on *F*, with *F* set to zero for negative *F*^2^. The threshold expression of *F*^2^ > σ(*F*^2^) is used only for calculating *R*-factors(gt) *etc*. and is not relevant to the choice of reflections for refinement. *R*-factors based on *F*^2^ are statistically about twice as large as those based on *F*, and *R*- factors based on ALL data will be even larger.

### Fractional atomic coordinates and isotropic or equivalent isotropic displacement parameters (Å^2^)

**Table:**

	*x*	*y*	*z*	*U*_iso_*/*U*_eq_	
C1	0.2014 (3)	0.2235 (2)	0.92052 (19)	0.0403 (5)	
C2	0.3302 (4)	0.1107 (2)	0.9031 (2)	0.0505 (5)	
H2	0.4576	0.0973	0.9285	0.061*	
C3	0.2687 (5)	0.0180 (2)	0.8478 (3)	0.0605 (7)	
H3	0.3518	−0.0596	0.8373	0.073*	
C4	0.0816 (5)	0.0425 (2)	0.8083 (2)	0.0573 (6)	
C5	−0.0465 (4)	0.1555 (3)	0.8239 (3)	0.0585 (6)	
H5	−0.1709	0.1703	0.7955	0.070*	
C6	0.0124 (4)	0.2463 (2)	0.8821 (2)	0.0512 (6)	
H6	−0.0739	0.3222	0.8955	0.061*	
C7	0.4960 (3)	0.4841 (2)	0.7696 (2)	0.0429 (5)	
C8	0.4849 (4)	0.6049 (2)	0.6737 (2)	0.0439 (5)	
C9	0.3391 (4)	0.6163 (2)	0.6007 (2)	0.0509 (6)	
C10	0.3493 (5)	0.7283 (3)	0.5067 (2)	0.0686 (8)	
H10	0.2534	0.7394	0.4565	0.082*	
C11	0.4987 (6)	0.8224 (3)	0.4870 (3)	0.0790 (9)	
H11	0.5026	0.8959	0.4237	0.095*	
C12	0.6422 (6)	0.8090 (3)	0.5598 (3)	0.0768 (9)	
H12	0.7427	0.8730	0.5460	0.092*	
C13	0.6361 (5)	0.7005 (3)	0.6529 (3)	0.0604 (6)	
H13	0.7331	0.6907	0.7023	0.073*	
C14	0.1835 (4)	0.5118 (3)	0.6165 (3)	0.0661 (7)	
H14A	0.0593	0.5222	0.6857	0.079*	
H14B	0.2528	0.4265	0.6336	0.079*	
H14C	0.1385	0.5197	0.5405	0.079*	
N1	0.3253 (3)	0.47376 (17)	0.87777 (17)	0.0434 (4)	
H1N	0.229 (4)	0.532 (2)	0.883 (2)	0.052*	
O1	0.0959 (3)	0.38337 (16)	1.08307 (14)	0.0554 (5)	
O2	0.4730 (3)	0.29640 (17)	1.02583 (16)	0.0581 (5)	
O3	0.6391 (3)	0.4000 (2)	0.75586 (18)	0.0674 (5)	
Cl1	0.00535 (19)	−0.07131 (8)	0.73539 (8)	0.0981 (4)	
S1	0.27927 (8)	0.34281 (5)	0.99049 (5)	0.0427 (3)	

### Atomic displacement parameters (Å^2^)

**Table:**

	*U*^11^	*U*^22^	*U*^33^	*U*^12^	*U*^13^	*U*^23^
C1	0.0376 (10)	0.0380 (10)	0.0435 (10)	−0.0018 (8)	−0.0103 (8)	−0.0029 (8)
C2	0.0508 (12)	0.0421 (11)	0.0553 (13)	0.0059 (9)	−0.0157 (10)	−0.0023 (9)
C3	0.0726 (17)	0.0405 (12)	0.0620 (15)	0.0046 (11)	−0.0101 (13)	−0.0089 (10)
C4	0.0726 (16)	0.0482 (13)	0.0489 (13)	−0.0211 (11)	−0.0081 (11)	−0.0063 (10)
C5	0.0505 (13)	0.0616 (15)	0.0664 (15)	−0.0146 (11)	−0.0199 (12)	−0.0062 (12)
C6	0.0405 (11)	0.0467 (12)	0.0681 (14)	0.0009 (9)	−0.0176 (10)	−0.0111 (10)
C7	0.0394 (10)	0.0468 (11)	0.0468 (11)	−0.0035 (9)	−0.0170 (9)	−0.0094 (9)
C8	0.0441 (11)	0.0461 (11)	0.0410 (10)	−0.0014 (9)	−0.0094 (9)	−0.0094 (8)
C9	0.0502 (12)	0.0609 (13)	0.0424 (11)	0.0084 (10)	−0.0136 (9)	−0.0144 (10)
C10	0.0818 (19)	0.0769 (18)	0.0469 (13)	0.0194 (15)	−0.0237 (13)	−0.0114 (12)
C11	0.112 (3)	0.0580 (16)	0.0533 (15)	0.0025 (16)	−0.0111 (16)	0.0036 (12)
C12	0.101 (2)	0.0567 (15)	0.0668 (17)	−0.0248 (15)	−0.0129 (17)	0.0003 (13)
C13	0.0670 (16)	0.0575 (14)	0.0588 (14)	−0.0147 (12)	−0.0176 (12)	−0.0082 (11)
C14	0.0479 (13)	0.095 (2)	0.0652 (16)	−0.0053 (13)	−0.0244 (12)	−0.0223 (14)
N1	0.0451 (10)	0.0387 (9)	0.0464 (10)	0.0006 (7)	−0.0129 (8)	−0.0073 (7)
O1	0.0640 (10)	0.0536 (9)	0.0427 (8)	0.0038 (8)	−0.0078 (7)	−0.0068 (7)
O2	0.0553 (10)	0.0641 (10)	0.0617 (10)	−0.0003 (8)	−0.0330 (8)	−0.0027 (8)
O3	0.0527 (10)	0.0709 (11)	0.0668 (11)	0.0169 (9)	−0.0106 (9)	−0.0015 (9)
Cl1	0.1417 (9)	0.0778 (6)	0.0845 (6)	−0.0445 (5)	−0.0243 (6)	−0.0269 (4)
S1	0.0446 (4)	0.0425 (4)	0.0418 (3)	0.0002 (2)	−0.0153 (2)	−0.0046 (2)

### Geometric parameters (Å, °)

**Table:**

C1—C2	1.379 (3)	C9—C10	1.399 (4)
C1—C6	1.385 (3)	C9—C14	1.493 (4)
C1—S1	1.760 (2)	C10—C11	1.376 (5)
C2—C3	1.378 (4)	C10—H10	0.9300
C2—H2	0.9300	C11—C12	1.373 (5)
C3—C4	1.381 (4)	C11—H11	0.9300
C3—H3	0.9300	C12—C13	1.371 (4)
C4—C5	1.377 (4)	C12—H12	0.9300
C4—Cl1	1.732 (2)	C13—H13	0.9300
C5—C6	1.376 (4)	C14—H14A	0.9600
C5—H5	0.9300	C14—H14B	0.9600
C6—H6	0.9300	C14—H14C	0.9600
C7—O3	1.205 (3)	N1—S1	1.6489 (18)
C7—N1	1.385 (3)	N1—H1N	0.819 (17)
C7—C8	1.491 (3)	O1—S1	1.4280 (17)
C8—C9	1.389 (3)	O2—S1	1.4243 (17)
C8—C13	1.394 (3)		
			
C2—C1—C6	121.3 (2)	C11—C10—C9	121.2 (3)
C2—C1—S1	119.81 (17)	C11—C10—H10	119.4
C6—C1—S1	118.90 (17)	C9—C10—H10	119.4
C3—C2—C1	119.5 (2)	C12—C11—C10	120.7 (3)
C3—C2—H2	120.3	C12—C11—H11	119.7
C1—C2—H2	120.3	C10—C11—H11	119.7
C2—C3—C4	118.8 (2)	C13—C12—C11	119.5 (3)
C2—C3—H3	120.6	C13—C12—H12	120.2
C4—C3—H3	120.6	C11—C12—H12	120.2
C5—C4—C3	122.1 (2)	C12—C13—C8	120.2 (3)
C5—C4—Cl1	118.6 (2)	C12—C13—H13	119.9
C3—C4—Cl1	119.3 (2)	C8—C13—H13	119.9
C6—C5—C4	119.0 (2)	C9—C14—H14A	109.5
C6—C5—H5	120.5	C9—C14—H14B	109.5
C4—C5—H5	120.5	H14A—C14—H14B	109.5
C5—C6—C1	119.3 (2)	C9—C14—H14C	109.5
C5—C6—H6	120.3	H14A—C14—H14C	109.5
C1—C6—H6	120.3	H14B—C14—H14C	109.5
O3—C7—N1	121.3 (2)	C7—N1—S1	125.08 (16)
O3—C7—C8	123.9 (2)	C7—N1—H1N	118.8 (19)
N1—C7—C8	114.76 (18)	S1—N1—H1N	115.6 (19)
C9—C8—C13	121.2 (2)	O2—S1—O1	119.23 (11)
C9—C8—C7	120.7 (2)	O2—S1—N1	110.43 (10)
C13—C8—C7	118.0 (2)	O1—S1—N1	103.74 (10)
C8—C9—C10	117.2 (2)	O2—S1—C1	108.92 (10)
C8—C9—C14	122.2 (2)	O1—S1—C1	109.13 (11)
C10—C9—C14	120.5 (2)	N1—S1—C1	104.33 (9)
			
C6—C1—C2—C3	−0.8 (4)	C8—C9—C10—C11	0.5 (4)
S1—C1—C2—C3	−179.73 (18)	C14—C9—C10—C11	−176.9 (3)
C1—C2—C3—C4	1.6 (4)	C9—C10—C11—C12	−0.2 (5)
C2—C3—C4—C5	−0.8 (4)	C10—C11—C12—C13	0.0 (5)
C2—C3—C4—Cl1	178.54 (18)	C11—C12—C13—C8	−0.2 (5)
C3—C4—C5—C6	−0.8 (4)	C9—C8—C13—C12	0.5 (4)
Cl1—C4—C5—C6	179.78 (19)	C7—C8—C13—C12	175.5 (2)
C4—C5—C6—C1	1.7 (4)	O3—C7—N1—S1	−7.6 (3)
C2—C1—C6—C5	−0.9 (4)	C8—C7—N1—S1	171.71 (15)
S1—C1—C6—C5	178.06 (18)	C7—N1—S1—O2	47.7 (2)
O3—C7—C8—C9	105.2 (3)	C7—N1—S1—O1	176.59 (17)
N1—C7—C8—C9	−74.1 (3)	C7—N1—S1—C1	−69.16 (19)
O3—C7—C8—C13	−69.9 (3)	C2—C1—S1—O2	−3.0 (2)
N1—C7—C8—C13	110.9 (2)	C6—C1—S1—O2	178.07 (18)
C13—C8—C9—C10	−0.7 (3)	C2—C1—S1—O1	−134.65 (18)
C7—C8—C9—C10	−175.5 (2)	C6—C1—S1—O1	46.4 (2)
C13—C8—C9—C14	176.7 (2)	C2—C1—S1—N1	114.98 (19)
C7—C8—C9—C14	1.8 (3)	C6—C1—S1—N1	−64.0 (2)

### Hydrogen-bond geometry (Å, °)

**Table:**

*D*—H···*A*	*D*—H	H···*A*	*D*···*A*	*D*—H···*A*
N1—H1N···O1^i^	0.82 (2)	2.15 (2)	2.925 (3)	157 (3)

Symmetry codes: (i) −*x*, −*y*+1, −*z*+2.
